# Untying the Gordian knot: remediation problems in medical schools that need remediation

**DOI:** 10.1186/s12909-018-1219-x

**Published:** 2018-05-31

**Authors:** Layne D. Bennion, Steven J. Durning, Jeffrey LaRochelle, Michelle Yoon, Deanna Schreiber-Gregory, Brian V. Reamy, Dario Torre

**Affiliations:** 1 Uniformed Services University of Health Sciences, 4301 Jones Bridge Road, Bethesda, MD 20814 USA; 2 University of Central Florida, 6850 Lake Nona Blvd, Orlando, FL 32827 USA

**Keywords:** Medical education, Academic remediation problems

## Abstract

This position paper discusses on-going academic remediation challenges within the field of medical education. More specifically, we identify three common contemporary problems and propose four recommendations to strengthen remediation efforts. Selecting or determining what type of remediation is needed for a particular student is akin to analyzing a Gordian knot with individual, institutional and systemic contributors. More emphasis, including multi-institutional projects and research funding is needed. Recommendations regarding language use and marketing of such programs are given.

## Background

Academic remediation is a near universal problem faced by medical schools and residency training programs. Studies suggest that the need for remediation is significant given 10% of medical students encounter an academic failure at some point during their training [1]. In response to this need, a high percentage of medical schools now offer some form of academic support for their students. Offering such services is laudable and necessary, but as many who have sat down across from a struggling student can attest, analyzing the contributing factors and deciphering a solution is akin to having a Gordian knot perched on your desk.

Curriculum reform efforts in the US are shifting to learner-based strategies, competency-based programs and respect for multiple modes of learning [2–5]. Similar curriculum reform efforts in Europe and the United Kingdom (UK) have been discussed and debated in recent years [6] including incorporation of the World Health Organization and World Federation of Medical Education (WHO/WFME) guidelines [7]. In addition, recruitment processes have widened their search for capable matriculants from diverse backgrounds [8–10] which requires changes in views of learning. All of these provide a unique opportunity to evaluate and revise aspects of the system.

Academic remediation within medical school programs is a topic of significant interest and has produced notable publications (e.g., Guerrasio [11] and Kalet and Chou [12]). However, remediation as a systematic process has not been overhauled from the traditional view that academic struggles are solely an individual problem which is handed off to remediation personnel. In this regard, we agree with Cleland and associates [13] and with Kalet, Ellaway and colleagues [14, 15] that remediation should be an explicit part of the structure of medical education with shared responsibilities rather than as an afterthought or “outsider” activity.

Nearly all medical schools invest in remediation resources, but most err in not having cross-talk between remediation specialists and instructors or curriculum managers at strategic points throughout students’ developmental process of becoming a physician. Instead remediation is relegated away from the core action of medical education; consigned to manage the ‘penalty box’ where struggling learners are sent after committing academic ‘fouls’. Without avenues of two-way communication between the remediation program and the larger educational program and without revising our view of remediation as ‘fixing’ struggling learners, remediation will remain, in our opinion, less effective and more costly to individual learners and to the school (see also [16]).

Cleland and associates article [13] as well as others have recently written about the disconnect between individual remediation needs and the larger educational system and have raised important associated questions. While Cleland and colleagues do offer some specific suggestions, overall they highlighted more problems and points for discussion rather than providing specific solutions. In this paper, we build upon their work and identify three common problems associated with the disconnect between the larger medical education process and remediation and subsequently propose four recommendations to minimize those problems.

### Problem 1. Struggling learners—Can the system find them early?

There are four potential sources for identifying struggling learners: pre-matriculation and demographic data, self-identification, student advancement or academic review committees, and the use of multiple-sourced, detailed, across-time performance data (portfolios).

Many risk contributors have been studied and are known before the first day of medical school. Some of those known risk factors are briefly reviewed below. Ferguson, James and Madeley [17] in their meta-analysis of studies involving students attending UK medical schools found that (1) women slightly out-perform men, and (2) identification as an ethnic minority tends to be a slight disadvantage. More recently, Woolf, Potts & McManus [18] found in their meta-analysis of UK physician training, wide-spread differences between white and non-white candidates. In addition, they found undergraduate grade point average (GPA) predicted only 23% of the variance in medical school grades, and learning style (e.g., Kolb’s convergers or the tripartite model’s deep and strategic) had weak correlations with academic performance (0.18 to 0.26). Donnon and associates’ [19] meta-analysis of Medical College Admissions Test (MCAT) score’s’ predictive validity of medical school performance found small to medium effect sizes (essentially zero up to 0.4) for various measures of later performance. Dyrbye et al. [20] found that students’ “serious thought” of dropping out of medical school was associated with demographic or event factors, e.g., being older in medical school (> 30 years old), having a child, being in the 3rd year of medical school, and self-identifying as an American Indian or Pacific Islander. As anticipated, they also found greater risk of academic problems was associated with significant psychosocial stressors such as major personal illness, major illness in a significant other, and divorce. No researchers have proposed that the above noted data points should be the sole identifiers of at-risk students. While these are known relationships, to the best of our knowledge, no researchers have combined known risk factors with concerning early performance data in an effort to identify struggling learners at the beginning of their academic training.

A second source for identifying struggling learners is self-referral. Only a small percentage of students self-refer to remediation resources. Combining 10 years of data, Guerrasio et al. [21] found approximately 7% of students were self-referred to a remediation program for medical students and professionals. Based on our experiences, many of these students tend to be high achievers whose drivers for self-referral include chronic anxiety about their performance. Thus seeking assistance or reassurance is not a new behavior for them. In our view, there is little change needed at the system level for this subset of struggling learners.

A third source for identification of an at-risk student is through a more formal process led either by faculty (Ellaway et al. [14]) or an academic or competency review committee. Typically, committees review a student’s situation following a critical academic or performance failure and then make recommendations to the school’s leadership regarding that particular student’s status within the school. While there is little question that the students who are reviewed by such committees are ‘at-risk’ learners, this action commonly occurs very late in the process. That is, remediation programs and experts sit by the “sidelines” until there has been a significant failure or a series of failures by an individual student. These institutional-level processes typically proceed slowly, are perceived as punitive by students and are designed to capture those students with the most serious academic or professional behavior problems, i.e., those who may be facing the possibility of disenrollment.

Stegers-Jager and colleagues [22] presented a different model from a Dutch medical school implemented recently which did include early identifications occurring at 4, 7 and 12 months into the preclerkship studies. Warnings were given at the 4- and 7-month time points, and academic probation was applied at 12-months. They did find more students participating in supportive services under this model (than the previous process). They found those students participating in one-on-one remediation services were significantly more likely to satisfactorily complete their preclerkship studies.

As for longer-term functioning of students reviewed by an academic review committee, Durning et al. [23] analyzed the performance of Post Graduate Year-1 (PGY1) residents comparing those who had been referred for academic review to those who had not. They found that being presented to the committee was associated with a higher risk of below average performance during PGY-1. However, they also found that the overall percentage of those students who interacted with the committee and had below average performance ratings during internship was low. The authors noted the need for more research including: (a) the timing and intensity of remediation efforts and whether either is associated with higher risk for later performance problems and (b) looking at the strength of the relationship with later performance comparing those students with primarily academic issues from those with primarily professional problems.

Similar to Kalet and colleagues [15, 24], we believe that as a student is identified later into the curriculum, the higher the likelihood of that student presenting with exhaustion, discouragement, and a lag in academic performance, which could have a potentially negative impact on the student’s ability to modify or attempt changes in their study process.

A fourth potential source for identification is the use of multiple-sourced, across-time performance of students analyzed with the goal of early identification of struggling learners. The notion of a learning or progress portfolio for medical students is certainly not new (e.g., [25–28].

Portfolios can be used as to means to comprehensively collect available performance data including academics. Recently, Kalet and Pusic [29] advocated use of a holistic and competency-based portfolio aggregating various scores and ratings from multiple sources. This process is currently in development at their program (New York University School of Medicine). Unfortunately, few details were provided regarding the management and design of this process. However, it is clear that the profile metrics are available to the student and their mentor for periodic review. One example table in their chapter listed 21 specific content “buckets” for various performance metrics such as histology, pathology, pharmacology, etc. as well as displaying for each student their performance as compared to their cohort for each content area in a box-and-whisker format. This is an example of an individualized and very detailed tracking system for students’ academic and professional performance and is in concert with our intended meaning for portfolio.

In Kalet and Pusic’s school, it appears such data remains with the student and their mentor and is not used by the larger system in identification of at-risk learners. Presumably, a student who consistently shows academic difficulties would be referred for additional assistance, but when or how the system interacts with or uses individuals’ data in the portfolio is not clear.

### Problem #2. Remediation—Which flavor works for which student?

Remediation, as a label, has been applied to a diverse set of processes including various skill-building presentations, tutoring, conceptual models, and one-on-one activities. With a few notable exceptions (see below), little is available which dissects the procedure and, most critically, details how to individualize remediation to meet the needs of a specific struggling learner. As noted by Winston [30] and others, simply teaching to pass the next exam does not work in the longer-term for struggling learners.

Over two thirds of medical school remediation programs make available services such as time management strategies, test taking techniques, note taking strategies, reviews of class exams, review of course content, modeling use of resources, and modeling problem-solving [31]. As noted by Cleland and colleagues a most noticeable downside to the above practices can be summed up as a systemic view that quick and easy fixes have the ability to address the problem of struggling learners [13]. Alternatively these generic services can ignore some of the individual contributors of a particular struggling learner. Some medical schools address the needs of at-risk students by providing pre-matriculation programs focused on enhancing study-skills and/or tutoring to promote early exposure to and learning of the material. Miller [32] described one such program which resulted in students modifying their anticipated study plan, but unfortunately no data was presented related to actual academic outcomes.

Remediation approaches applied in medical education research have been categorized into four models: analytic, developmental, synthetic and competency-based [33]. Unfortunately, such models have been found to explain little of the outcome variance whether referring to clinical skills development, basic science knowledge or professionalism [16, 34–37].

Winston [30] discussed an in-depth, year long, multi-pronged, intensive program for students repeating a portion of the curriculum involving regular faculty-facilitated, small group work with integration of study skills and presented data indicating their success. Sayer et al. [38] described a small, reportedly successful program in a UK medical school specifically targeting academic issues with lengthy one-on-one tutoring, a collaboratively designed remediation plan and a careful individualized assessment process.

An alternative approach is a listing of potential types of learner deficits (“diagnoses”) such as difficulties in fund of knowledge, clinical reasoning, history-taking, physical examination or professionalism [39]. Guerrasio [11] adds the following additional remediation ‘diagnoses’: time management and organizational skills, interpersonal skills, communication, practice-based learning, system-based learning and mental well-being.

Beyond descriptive summaries and looking at scattered outcome metrics, little research has focused on how to build an effective and individualized process to provide successful remediation. Ascertaining what works and doesn’t work in remediation is difficult. Guerrasio [11] has perhaps one of the most detailed and practical discussions based on their remediation process at University of Colorado School of Medicine and she diligently references many sources supportive of particular processes. Kalet and Chou [12] also document many practical aspects and viewpoints related to conducting remediation with individual students. As these authors and others (e.g., [13, 40]) note most research on medical education academic remediation is focused on a retake of a failed examination and provides little basis for determining of what types of support are needed for which students or what type of remediation is most helpful. Kalet, et al. [12] rightly note “as yet [there is] little evidence supporting how and why remediation in medical education works” (pg 24).

A second complication in individualizing effective remediation is the varied background of staff who are tasked to create or manage remediation programs. A survey of 134 medical schools found that less than half of the staff who were responsible for remediation in medical schools had graduate degrees in education, learning or in enhancing individual behavioral change [31]. Over one third of these staff members reportedly had no particular training nor prior experience in providing academic support. Similar to above, this data suggests remediation at the graduate level is a young field with the added disadvantage of not having a solid basis in theory or discipline.

A third complication, as identified by Guerrasio [11], Sayer et al. [38] and Mcloughlin [41] focuses on the numerous contributors to academic difficulties. For a particular student, the number of contributors to academic struggles is typically multiple per struggling learner and many are related to the student’s background experiences and/or to current vulnerabilities within their social and family settings. Thus, teaching specific skills, e.g., time management or the notion of spaced learning, is often an inadequate response for at least a portion of struggling students. Cleland and associates [13] also highlight the need to recognize pressures from institutional culture as well as individual student backgrounds.

Durning and associates [33] take a different approach. Rather than attempting to match remediation strategies to a specific student or their learning difficulties, they took an empirically and theory-based application of a self-directed learning process which is generic to topic and context and adapted it for struggling medical learners. This model, Self-Regulated Learning—Microanalytic Assessment and Training (SRL-MAT), is a problem-solving approach which functions as a three-phase cyclical loop and is applicable across learning situations. Specifically the three cycles are: (a) Forethought including strategic planning and motivational factors, (b) Performance involving metacognitive monitoring, self-testing and self-control and (c) Self-Reflection about performance such as causal attribution, self-evaluation and satisfaction. These skills are intended for the application of immediate to longer-term work products. Notably, this model is based on a solid foundation in educational research with data showing successful learning outcomes from multiple learning contexts and has been successfully utilized in improving the diagnostic reasoning of medical students [42].

Fourth, rarely is there any formal assessment of the struggling learner, beyond an unstructured interview, to objectively measure their academic and cognitive strengths and weakness. While many remediation programs include an interview in their process, what is meant by ‘an interview’, the degree of structure and coverage for that interview and how that information is used is a glaring hole.

A welcome exception is Yellin’s [43] work which discusses the issues associated with formal cognitive assessment, determining strengths and weaknesses and if testing supports it, applying diagnosis of learning disabilities for medical students. He advocates for the use of broad-based cognitive testing to ascertain struggling medical students’ relative strengths and weaknesses within six neurodevelopmental constructs (attention, language, memory, temporal-sequential ordering, spatial ordering and higher-order cognition) to individualize the remediation plan. His process outlines the use of objective testing to create a cognitive profile used to help students better understand their cognitive and learning relative strengths and weaknesses. Based on the individual profile, the remediation team develops a tailored learning plan or a “management by profile” plan. When testing is not utilized, some academic weaknesses may not be recognized or addressed such as subtle reading comprehension problems, a need for better writing skills or below average clinical reasoning skills [44]. A downside to the above process is the high cost and time needed for formal assessment as well as a specialist (e.g., neuropsychologist) who is familiar with the necessary testing tools and, critically important, is willing to frame an evaluation within the context of graduate medical education (rather than simply using general population or age-based norms).

At the individual student level, as Mcloughlin [41] and Yellin [43] noted many students use the same strategies which produced success for them in their secondary and post-secondary educational endeavors. When faced with performance difficulties in medical school, these students often redouble their use of those same well-used strategies. Finding a new strategy is time-intensive and few medical students take the risk of potentially wasted time in trying new strategies. Medical students, almost by definition, are highly successful learners across decades of learning and adaptation to many environments. For these students any serious academic problem, not to mention consistent academic failures, is an unexpected and foreign hardship which they have no experience managing or learning from in productive ways. For the student in the throes of early professional identity development as a physician, serious academic problems can challenge their resiliency, motivation and career goals.

Fifth, research which incorporates demographic and cultural differences as covariates beyond gender and age are lacking. There are many who advocate the need for diversity and minority representation in the medical professional [45–49]. Difficult issues such as communication within cross-cultural supervision and mentoring of medical trainees has been addressed [50]. Little if any attention is focused on any empirical remediation investigations which also included broader diversity issues in the medical literature.

Sixth, there is scant recognition in the remediation literature of the difficulties all learners face when attempting to change their well-learned and previously servable strategy. The most commonly used skills deficit model in remediation asks students to change strategies and habits which have been personally effective for years and successfully used for thousands of hours. Changing deeply ingrained behaviors is difficult, stressful, and early attempts can be easily abandoned before experiencing success with an alternative strategy.

A final consideration is acknowledgement that a massive percentage of students are masterfully successful in adapting to the challenges of graduate medical education. Unfortunately, almost nothing is known about how high performing versus ‘below average’ medical students adapt. Information regarding how ‘super’ learners in medical school change, adapt and accomplish what they do is lacking in the educational literature. Discovering their successful adaptive strategies may add additional tools to the remediation process.

### Problem 3. Stigma—It continues to interfere

At the individual student level, most medical students are historically unacquainted with academic difficulties [13] and are reluctant to come forward and ask for additional assistance [41, 51, 52]. In addition, some students have a bias in their perceptions of what causes these academic problems. For example, Cleland and associates [40] found that 4th year medical students in a UK medical school with below average performance tended to blame external factors, believe that their most recent failure was an isolated event and believe that faculty did not offer the appropriate assistance (when in fact multiple messages to aid these same students had been sent).

At the institutional level, few would find overt bias against students needing additional assistance. However, there seems to be a subtle belief which may affect how leaders and universities perceive remediation programs. Specifically, a view that students with early academic difficulties will continue to struggle throughout their professional development, leading to a future of mediocre doctoring skills. For example, Cleland et al. [40] stated “weak medical students go on to become weak doctors” and cited two references [53, 54]. Both of the cited sources highlight professional behavior problems and/or communication problems between team members. Neither source is focused on the relationship between early academic difficulties and later “poor” doctoring. For example, the first source, Papadakis and associates [53] concluded that low MCAT scores and low grades during the first two years of medical had “only ¼ the risk” of subsequent disciplinary action (as compared to the risk faced by student with early professional behavior problems). Based on the numbers Papadakis and colleagues presented, a student with an academic failure of a medical course during the 1st or 2nd year of medical school, had only a 7% risk of subsequent disciplinary action by state board. In contrast, those with documentation of unprofessional behavior during medical school, had more than three times that risk (26%) of subsequent disciplinary action by state board. In a similar study Papadakis [55] did not find academic metrics (e.g., undergraduate GPA, lower quartile MCAT performance, not passing one or more medical school courses) were a significant predictor using logistic regression modeling of later professional behavior problems. A statistically significant difference was noted between the undergraduate GPA of the group with later disciplinary actions and other graduates (3.3 vs. 3.4), but this is not a meaningful difference at the level of individual students nor was this variable a significant predictor in the logistic regression results.

Similarly, a study of over 3000 Canadian physicians found no statistically significant relationship between a score indicative of knowledge competency (score from a traditional written exam with 450 multiple choice questions (MCQs)) and future patient complaints against the physicians [56]. Guerassio [21] in a study of over 150 struggling learners spanning from undergraduate to graduate medical education, found only professionalism issues were a significant predictor of later probation, and that medical knowledge was not a predictor. In Santen and associate’s [57] study of students identified by student academic promotions committee either through academic issues or behavioral problems, approximately 4% of those students later had state medical board actions taken against them for unprofessional behavior. All of the students in their sample who had professional behavior difficulties also had academic issues. Scrutiny of Santen’s published summary of their data suggests it is possible students who only had academic issues and were not also identified as having behavioral problems, were not later disciplined by state boards for unprofessional behavior (however, this is uncertain based on how they presented their data). LaRochelle et al. [58] found evidence that pre-clerkship performance in clinical reasoning (small group exercises, multiple choice examination focused on clinical reasoning and Objective Structured Clinical Examination (OSCE) performance) did have small, but significant predictive value of resident ratings of medical expertise by faculty.

In addition, there are studies that suggest early academic performance or even board examination scores are unrelated to other important non-knowledge-based aspects of physician care. For example, Hojat et al. [59] found no relationship between scores on United States Medical Licensing Examination (USMLE) Step 1 examination and empathy scores. No studies were found directly linking early low academic performance (pre-med school or pre-clerkship) as a primary or substantial contributor to later poor quality of doctor-patient relationships, diagnostic errors, treatment errors, or patient satisfaction. This view is perhaps the flip side of a long tradition of high academic performance being the principal gatekeeper for entry into certain specialties; academic performance is seen as a proxy for doctoring capacity or aptitude (see Prober et al. [60] for additional discussion of the pros and cons of using Step exam scores as primary selection variable of residents).

An additional factor to be identified when considering the subtle institutional bias against remediation is that much of academic remediation literature borrows heavily from the universally familiar medical model, that is, find the diagnosis and once the diagnosis is known, that diagnosis ‘determines’ treatment. While this view obviously has heuristic usefulness, the extent that this viewpoint should be extended to remediation of individual students is unclear. Given this model is steeped in the assumptions of disease, pathology processes and (body) system failures, it is easy to imagine how, even if unintentionally, promotion of this view can carry negative assumptions. For example, even if a student is identified as a struggling learner, this does not necessarily mean that the student has a fundamental flaw or set of deficits that would negatively impact that student’s functioning as a physician. Much of medical education support services, e.g., teaching a specific and presumed missing skill, are based on the above unstated assumption that the pace and rigor of medical school exposes a pre-existing skills deficit. That is, the student performed lower on an examination than expected, therefore the student has an academic or study skills deficit and/or a motivational problem.

#### Potential solutions

Next we turn to potential solutions to deal with these challenges that face the medical education community. Here, we list four recommendations.

#### Recommendation #1. Use systematically captured data, across-time, multiple-sourced information with one of the goals being early identification of struggling learners

The concept of and use of portfolios in medical education is certainly not new. However, many uses of portfolios cited in the medical education literature appears to be a collection of student performance datum without organization into competency domains and without ways to represent growth over time within those competency domains. For example, Chertoff and associates [61] surveyed 71 Liaison Committee on Medical Education (LCME) accredited medical school. Approximately half reported use of portfolios, but less than 10% of total respondents noted they felt their portfolio process included a visual display of competency growth. Other recent publications noting uses of portfolios are within particular modules or rotations, e.g., Sanchez Gomez [62] or within particular aspects of training such as simulation centers [63]. While helpful, these do not provide indications of overall growth or, in the case of remediation, lack of individual progress.

We advocate for the use of a portfolio as an across-the-curriculum, integrated longitudinal assessment tool to guide and help the remediation process such as discussed by Van Tartwijk et al. [64]. A comprehensive portfolio includes an organizational schema such that various assessments are gathered from multiple sources and placed into one or more general competency domain or “bucket” as labelled by Kalet’s group [29]. It allows learner and teachers to support students’ learning and closely assess students’ progression [65].

Specifically, we recommend a comprehensive portfolio of all performance data (knowledge-based, group performance, OSCE-type performance, written reports of patient encounters, self-assessment of professional growth, ratings or commentary of videotapes of leaning interactions, etc.) be collected. Further that this collected data be made available to the student as well as to selected key individuals on the academic team (e.g., the remediation team and those charged with tracking performance and with early identification of struggling learners). Importantly, we recommend that at least one reviewer of student portfolio data should be tasked with taking a longitudinal view of the learner’s progress particularly within competency ‘buckets’ as discussed above. That is, the longitudinal reviewer is not as concerned with rating or grading a student performance related to current courses, but is to monitor, over time, for marginal performance within a competency area. Thus, remediation is not just studying for the next exam, but also analyzing developmental patterns. Furthermore the concept of portfolio-based monitoring and learning may be particularly important in the remediation process [25, 27]. The use of a portfolio that contains a large amount of longitudinal information about learning interactions and learners’ performance across settings and topical domains. Thus, such information may be particularly useful in developing and implementing remediation plans for some students which otherwise would have gone unrecognized. In addition, once such a longitudinal portfolio process is put into place, the school could eventually analyze previous years of data to potential identify school-specific performance markers within early curriculum experiences which, when paired with known pre-matriculation risk factors, could help identify students at risk for later serious academic problems.

The use of performance portfolio raises the concern of ‘forward-feeding’ performance problems to subsequent faculty or clerkship directors. This has been debated in the literature [16, 24, 66–68]. Clearly there are privacy concerns and other issues to consider. Much of above dialogue centered upon who should have access to such information including clerkship directors and/or faculty and discusses the potential for negative (and positive) biases. Our opinion is that there should exist a separation of the processes which determines official student status (typically a slow-moving, large group-based process triggered by significant failure or string of failures) and the remediation team (desirably a faster-moving, dynamic process). Ideally this latter team is a small group, is closer to ‘real time’ in its tracking of student performance and is skilled in its identification of struggling students. Its function falls within student advocate services and it should engage early and often with the struggling student. We strongly recommend those involved in reviewing and using that data for early identification of struggling learners be either separate from or not have a formal vote into procedures which determine student status within the program.

#### Recommendation 2. Encourage and fund research investigating the complex processes of medical education at the level of individual change including a variety of contributors

Directly assessing remediation is difficult. However, finding active change agents is critical. In medical research, we sensitively delve into individuals’ complex personal lives to probe aspects of cancer, diabetes, degenerative diseases, etc. and compare treatment alternatives. However, we are slower to apply similar rigor, energy and funding to medical remediation. Critics may argue academic difficulties during medical education is not a disease nor a pathology which affects the population at large or may consider it a non-problem as only affects a small percentage of students and thus doesn’t need additional funding or focus. In our view, this perspective is out-of-touch with the current directions and values of medication education.

Acquiring large numbers of struggling learners to study is difficult at the institutional level, but is less so if collaborative multi-institutional studies are implemented. Implementation of work might involve consortiums across a small number of medical schools, shared development of performance portfolios, identification of similar performance metrics, as well as integrating and analyzing de-identified performance data across institutions.

In addition, many remediation programs appear to function as independent islands and offer services that are based on the experience and skillsets of individual remediation specialists. More coordination and sharing of ideas, resources, and processes could help more remediation forward as well as generate data to answer hypothesis-driven questions concerning optimum remediation methods.

#### Recommendation 3. Use of De-stigmatizing processes and language

The traditional ‘diagnosis➔treat’ view of remediation is problematic. No heuristic is perfect. But perhaps a better model for conceptualizing struggling learners within medical education is that of athletic conditioning for a team of gymnasts or decathlon athletes. Each athlete has years of experience, a history of many high-level performances, but each athlete, each coach and the institution understand qualification is at an individual level and must occur in a variety of skillsets or performances. Every athlete experiences strong events and weak performances. For these high-functioning athletes, conditioning (or remediation) is not about discovering a pre-existing flaw. The foundation of conditioning is a careful and detailed analysis of recent performances, identification of relative strengths and weaknesses and then designing targeted exercises to enhance performance, dedicated time to build up weaker skills as well as developing and practicing strategies for key performances.

We recommend reducing the use of terms such as remediation, diagnosis, deficits, etc. A more useful articulation may be using terms such as ‘not meeting a qualification standard’. While cumbersome, such phrasing suggests a student did not achieve their maximum potential for a particular performance event rather than the implication that the student, as a holistic organism, is dysfunctional, incapable or lacks an essential skillset which bars them from a success within the medical school context.

We are working with highly qualified and gifted students who are being stretched to their near maximum capacity for years at a time. Thus, we recommend terms such as *academic conditioning, performance coaching, learning strengths and weaknesses (or cognitive and learning profile),* and *adaptive strategies*. Perhaps even terms such as “ath-learner” or “aca-lete” should be considered. While this last suggestion is somewhat tongue-in-cheek, the underlying notion detailed above is not. We are not simply recommending a change in the label. The recommendation is to move the culture away from terminology which inherently supports an assumption of pathology and deficit and instead use language which recognizes: (a) there are complex factors impacting performance and (b) these students are above average learners operating in a very high-demand environment.

#### Recommendation 4. Encourage self-identification and normalize help-seeking

A one-time lecture simply reiterating above messages is unlikely to provoke change in student’s help seeking behavior (see [69] for example). Friedman and associates [70] found, in their efforts to reduce de-stigmatizing attitudes toward mental health usage, that a lecture-based format was perceived as patronizing by medical students. However, they did find that students were more receptive to testimonials from individuals who were using mental health-related services. In recent work, Cleland and colleagues [13] discussed the importance of the institutional culture affecting help-seeking behavior and they offered a few specific suggestions, e.g., asking the student their views of contributors to academic difficulties, working in partnership with struggling learners, and the staff role-modeling self-care.

A useful option to normalize help seeking over time may be for medical schools to annually ask for volunteers among graduating students and faculty to record brief personal audio or video ‘testimonials’ of healthy help-seeking options they utilized during medical school (each volunteer choosing to specifically identify themselves or not). A brief suggested outline or reminders (e.g., list of common hurdles) might assist volunteers to address known barriers, e.g., confidentiality, worries about effect on career as a student, being reluctant to self-identify as having additional needs, etc. [71, 72]. It may be appropriate to instruct the volunteers to not individually name less-than-helpful local individuals or providers. Obviously, the volunteers would not be expected to disclosure personal details of their difficulties, but are asked to provide only a generic reference to how stressors impacted them personally while in medical school and then focus on helpful options they experienced which helped them not only manage, but succeed. These personalized help-seeking declarations could then be made electronically available to successive cohorts who could access them as needed through their schooling via networks internal to the medical school. Making it known that certain individuals within leadership and faculty of the medical school are willing to provide such testimonials may encourage graduating students to do the same.

In addition, brief messages from the current school leadership supporting appropriate help-seeking by students being available within the same venue may be helpful as well. Also, as part of school leadership messages, referencing studies noting the percentage of medical students who do seek assistance can be a normalizing factor. For example, Dyrbye and associates [71] found approximately 40% of medical students annually seek out formal or informal supports.

Third, we recommend applying the well-established medical model of consultation to academic issues and struggling learners. Again, leadership and supervisor messaging is critical. That is, rather than telling students with academic problems they are in need of an evaluation to detect and diagnosis weakness and deficits, we recommend referring to need for additional resources as an academic consultation. As physicians, providers are expected to recognize when specialty care or evaluation is needed particularly when standard treatments are not effective. Why should not a similar attitude be fostered from the beginning of their medical training? The message should be, “If you have adequately applied the recommended ‘treatments’ to your studies and those are not effective, it is time to obtain a consultation!”.

There are, of course, limitations to our discussion and proposals. All of these issues are challenging and no solution will fit all situations or schools. Along with research investigating active ingredients of successful remediation, research into the links between remediated students and longer-term outcomes is needed.

## Conclusions

This position paper discusses some on-going problems within the field of medical education, identifying four contemporary problems and proposing four recommendations to strengthen remediation efforts. Earlier identification and assistance to learners “not meeting qualification standards” has been discussed by many, but is rarely implemented in a systematic manner. We recommend the use of a professional performance portfolio organized by competency domains and include techniques to show learner progress over time as a way of addressing this long-standing problem. Curriculum revision offers opportunities to incorporate programmatic approaches to remediation. Selecting or determining what type of remediation is needed for a particular student is a Gordian knot. More emphasis, including multi-institutional projects and research funding is needed. Remediation as an effort continues to be a seen as a necessary evil within the larger mission of medical education as opposed to a program that strengthens and conditions individual learners, and in turn the institution and ultimately medical practice. Recommendations regarding language use and marketing of such programs are given.

## Abbreviations

GPA: Grade Point Average
LCME: Liaison Committee on Medical Education
MCAT: Medical College Admissions Test
MCQ: Multiple Choice Questions
OSCE: Objective Structured Clinical Examination
PGY1: Post Graduate Year 1
SRL-MAT: Self-Regulated Learning—Microanalytic Assessment and Training
UK: United Kingdom
USMLE: United States Medical Licensing Examination
WFME: World Federation for Medical Education
WHO: World Health Organization
